# Identification and Genetic Analysis of a Factor IX Gene Intron 3 Mutation in a Hemophilia B Pedigree in China

**DOI:** 10.4274/tjh.2013.0275

**Published:** 2014-09-05

**Authors:** Dong-Hua Cao, Xiao-Li Liu, Kai Mu, Xiang-Wei Ma, Jing-Li Sun, Xiao-Zhong Bai, Chang-Kun Lin, Chun-Lian Jin

**Affiliations:** 1 Hospital of PLA, Aristogenesis Center, Shenyang, China; 2 Shenyang Women’s and Children’s Hospital, Assisted Reproductive Technology Laboratory, Shenyang, China; 3 Zibo Maternal and Child Health Hospital, Genetic Disease Laboratory, Zibo, China; 4 China Medical University, Department of Medical Genetics, Shenyang, China

**Keywords:** Hemophilia B, Factor IX, mutation, Intron 3, mRNA splice site

## Abstract

**Objective:** Hemophilia B is caused by coagulation defects in the factor IX gene located in Xq27.1 on the X chromosome. A wide range of mutations, showing extensive molecular heterogeneity, have been described in hemophilia B patients. Our study was aimed at genetic analysis and prenatal diagnosis of hemophilia B in order to further elucidate the pathogenesis of the hemophilia B pedigree in China.

**Materials and Methods:** Polymerase chain reaction amplification and direct sequencing of all the coding regions was conducted in hemophilia B patients and carriers. Prenatal diagnosis of the proband was conducted at 20 weeks.

**Results:** We identified the novel point mutation 10.389 A>G, located upstream of the intron 3 acceptor site in hemophilia B patients. The fetus of the proband’s cousin was identified as a carrier.

**Conclusion:** Our identification of a novel mutation in the F9 gene associated with hemophilia B provides novel insight into the pathogenesis of this genetically inherited disorder and also represents the basis of prenatal diagnosis.

## OZET

**Amaç:** Hemofili B, X kromozomu üzerinde Xq27,1’e lokalize faktör IX genindeki koagülasyon defektleri nedeniyle oluşur. Hemofili B hastalarında yaygın moleküler heterojenite gösteren ve geniş bir dağılımı olan mutasyonlar tanımlanmıştır. Çalışmamız Çin’deki bir hemofili B ailesindeki patogenezi açığa kavuşturulmak için hemofili B’nin genetik analizi ve prenatal tanısını amaçlamıştır. 

**Gereç ve Yöntemler:** Hemofili B hastalarında ve taşıyıcılarda tüm kodlanan bölgelerin polimeraz zincir reaksiyonu ile amplifikasyonu ve direkt dizileme yapılmıştır. Probandın prenatal tanısı 20. haftada yapılmıştır. 

**Bulgular:** Hemofili B hastalarında yeni bir nokta mutasyonu olan 10,389 A>G’nin intron 3’ün alıcı bölgesinin yukarı akımında bulunduğunu tanımladık. Probanın kuzeninin cenininin de taşıyıcı olduğu bulundu. 

**Sonuç:** F9 geninde hemofili B ile ilişkili yeni bir mutasyonu tanımlamamız genetik olarak kalıtılan bu hastalığın patogenezine yeni bir açıklama getirmiştir ve prenatal tanının temelini temsil etmektedir.

## INTRODUCTION

Hemophilia B (HB) is an X-linked inherited disorder resulting from deficiency in the blood coagulation factor IX (F9) caused by mutation of the factor IX gene (F9; GenBank accession number K02402.1). F9 is located at Xq27.1 and comprises 8 exons encoding 6 functional domains: prepropeptide, the Gla domain, epidermal growth factor (EGF)-1 and EGF-2 domains, the activation domain, and the catalytic domain [1]. The gene spans approximately 31 kb with an mRNA transcript of 2803 bases encoding a protein of 461 amino acid residues. The F9 gene and protein share considerable sequence homology and near identical structural organization with the coagulation vitamin K-dependent serine proteases factor VII, factor X, and protein C. However, a wide range of mutations, showing extensive molecular heterogeneity, have been described in patients affected by HB of varying severity [2].

In 2011, we ascertained a HB pedigree from Liaoning Province, China. In this study, we conducted genetic analysis and prenatal diagnosis of HB in order to further elucidate the pathogenesis of the HB pedigree and help a pregnant woman deliver a healthy baby.

## MATERIALS AND METHODS

**Proband**

At 6 months of age, the proband (male, born on 20 February 2008) presented with pelioma, cyanotic bumps, subcutaneous bleeding, and knee swelling following crawling movement. The child was diagnosed as factor IX-deficient (test results: F9 coagulum activity 1%) by the Department of Hematology of Beijing Union Hospital in May 2011. Currently the child exhibits swelling of both knee joints and limited mobility, and he has been treated with monthly doses of factor IX.

**Pedigree**

**Figure 1:**
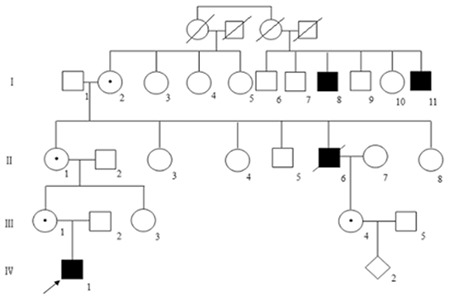
Family pedigree. Solid symbols indicate affected individuals; circles represent females and squares represent males; open symbols indicate healthy individuals; circles with black dots indicate carriers. The proband is labeled with an arrow.

**Figure 2:**
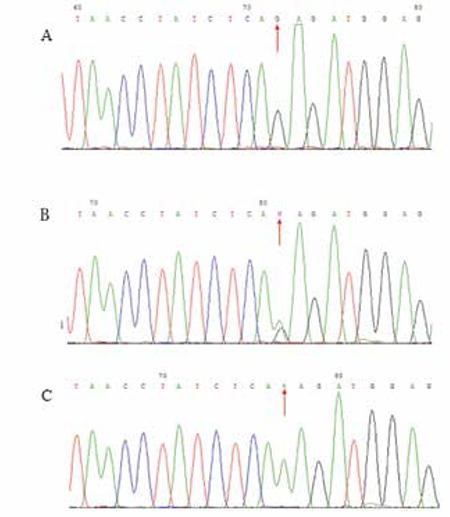
Sequencing maps of the F9 gene in patients and carriers. A, the proband; B, the proband’s mother (carrier); C, a normal control. Arrows indicate mutation loci.

The identified HB pedigree comprises 4 patients (Figure 1,2). I 8, I 11, and II 6 exhibited similar symptoms as the proband. III 4 was pregnant (20 weeks).

**DNA Isolation and DNA Analysis with PCR**

Genomic DNA samples of patients (I 8, I 11, IV 1), 4 obligate carriers (I 2, II 1, III 1, III 4), and 100 healthy adults from our center were isolated from peripheral blood leukocytes using a DNA extraction kit (Tiangen, Beijing, China). The F9 exons were amplified in a total of 7 reactions using the primers and polymerase chain reaction (PCR) conditions shown in Table 1. Sequencing was performed using the BigDye® Terminator v3.1 cycle sequencing kit (Applied Biosystems, Foster City, CA, USA) and analyzed with the ABI 3130 Genetic Analyzer. Exons 1 to 8 of the F9 gene were sequenced in all patients. The F9 sequence was used as a reference and mutations were numbered accordingly [3].

**Amniotic Fluid Culture Conditions and Treatment**

A third-generation member of the pedigree (III 4), a 25-year-old female, was pregnant (20 weeks). She was identified as a carrier by sequencing analysis and the couple received genetic counseling at the Medical Center of the No. 202 Hospital of the People’s Liberation Army, China. The genetic development of HB and the probability of the presence of this gene in the fetus, as well as the consequences if the child was affected by the disorder or identified as a carrier, were explained in detail. Amniocentesis and amniotic fluid culture were then carried out at the parents’ request. DNA analysis was performed as described.

**FIX mRNA Analysis**

The F9 gene is expressed as a 2.8-kb mRNA. Elucidation of mutations in the F9 gene is important in understanding how such mutations affect the function of the F9 protein. To investigate this issue, RNA was extracted from the peripheral blood of patients and normal controls using the Blood RNA Kit (Tiangen) according to the manufacturer’s protocol. Using the Reverse Transcription Kit (Promega, Madison, WI, USA), cDNA was generated according to the manufacturer’s instructions. Intron 3 was amplified with 2 pairs of primers containing the 5’-end GT donor splice site and the 3’-end AG acceptor splice site, which were designed using Primer5 software. The products were 438 bp and 213 bp in length, respectively. Amplification of the β-actin gene (the β-actin is specific to the cDNA) was included as a reference (626 bp). Details of the primers and PCR conditions are shown in Table 2. The PCR products were ligated into the TA cloning vector for sequencing as described.

**Sequencing of Intron 3**

A pair of primers for amplification were designed using Primer5 software to amplify intron 3. The upstream primer (5’-TTGAAGAAGCACGAGAA-3’) is located in exon 2 while the downstream primer (5’-AACAACCCGAGTGAAGT-3’) is located in exon 6. The target fragment size in normal human cDNA is 473 bp. PCR amplification was performed using patient and control cDNA as templates and sequencing was conducted as described.

## RESULTS

**Genetic Analysis**

Screening for molecular differences in the F9 gene was conducted by sequencing of genomic DNA in 3 patients, 4 carriers, and 100 healthy adults. We identified a novel point mutation at position 10,389 (A>G), located upstream of the AG splice site between intron 3 and exon 4. All carriers were identified as heterozygotes and the mutation was not detected in the 100 healthy adults.

**Prenatal Diagnosis**

The fetal DNA was screened for prenatal diagnosis at the request of the mother (carrier) using the same method. The results identified the fetus (IV 2) as a heterozygote for this mutation, thus confirming the child to be a carrier for HB. The prenatal protocol included 2 counseling sessions, psychosocial evaluation of the couple, and an obstetric assessment.

**Study of HB Pathogenesis**

**Figure 3:**
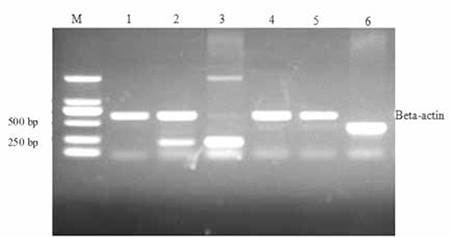
PCR amplification of the 3’-end and 5’-end of factor F9 intron 3. Lanes 1, 2, and 3 represent the 3’-end of intron 3 amplified from normal human cDNA, patient cDNA, and normal human DNA as templates; Lanes 4, 5, and 6 represent the 5’-end of intron 3 amplified with normal human cDNA, patient cDNA, and normal human DNA as templates. The β-actin fragment was not amplified in Lanes 3 and 6 because β-actin cannot be amplified from DNA as it is specific to the cDNA. Lane M is a size marker.

The target fragment (213 bp) containing the 3’-end AG acceptor splice site at the intersection of intron 3 and exon 4 was successfully amplified from normal human DNA (Lane 3 of Figure 3) and patient cDNA templates (Lane 2 of Figure 3). However, the target fragment was not amplified from the control cDNA template (Lane 1 of Figure 3), suggesting that intron 3 and exon 4 were correctly spliced and there was no residue of intron 3 in the cDNA of the control cDNA. The correct sequences of PCR products confirmed the presence of a residual part of intron 3 in the cDNA of the HB patient.

The target fragment (438 bp) containing the 5’-end GT donor splice site of intron 3 at the intron 3/exon 3 intersection of the F9 gene was not amplified from the patient (Lane 5 of Figure 3) or control (Lane 4 of Figure 3) cDNA templates. However, the target fragment was successfully amplified from normal human DNA (Lane 6 of Figure 3) and the correct sequences were confirmed. This demonstrated the functional splicing activity of the 5’-end GT donor splice site of intron 3.

**Sequencing of Intron 3**

The target fragment was successfully amplified from normal human cDNA templates and the correct PCR products were confirmed by sequencing. The target fragment was not amplified from HB patient cDNA templates.

## DISCUSSION

HB occurs in approximately 1 in 30,000 male live births [4] and this rate is not significantly affected by the ethnicity of the population. In all cases, HB is caused by mutation of the F9 gene, although mutation analyses of different cohorts have demonstrated extensive heterogeneity [5,6,7]. Mutations of this type are generally thought to include point mutations, missense mutations, nonsense mutations, mRNA splice site mutations, deletions, and rearrangements/inversions [1]. However, this study of a HB pedigree in China identified a novel mutation (10,389 A>G) in intron 3 of the F9 gene, located upstream of the AG splice site between intron 3 and exon 4.

The third base of the upstream 3’ acceptor splice site of the F9 gene intron 3 (-3 A>G) was identified as a heterozygous mutation locus in the proband’s mother (III 1) as well as the maternal grandmother (II 1), great-grandmother (I 2), and maternal first cousin (III 4), all of whom were carriers. The fetus (IV 2) of III 4 was identified as a carrier by prenatal diagnosis. The baby girl was delivered normally and normal F9 activity was confirmed.

Intronic splice sites are critical for maturation of pre-mRNA. Therefore, we investigated the presence of splice site mutations in the F9 gene at the mRNA level by RT-PCR analysis. It can be speculated that the (-3 A>G) mutation may lead to failure of normal intron 3 splicing. We propose that the mutated splicing site leads to abnormal transcription of DNA to mRNA. Consequently, the abnormal mRNA results in the synthesis of abnormal F9 protein, thus causing patient morbidity. It is also possible that abnormally spliced mRNA cannot enter the cytoplasm, resulting in the absence of the translated protein. Thus, we hypothesize that the (-3 A>G) mutation at the base upstream of the intron 3/exon 4 AG splice site in the patient in the pedigree results in aberrant splicing of the F9 mRNA. This accounts for the presence of partial residual sequences of intron 3 in patient cDNA, although it is unclear how long these sequences are maintained in patient cDNA. Amplification of this region was not achieved from patient cDNA even with the use of multiple primer pairs. Several factors may account for this: 1) no target fragment was amplified by PCR in the presence of excessive residual intron 3; 2) amplification is hindered owing to a high GC content and repetitive sequences in the introns; 3) the F9 gene in the patient cDNA is cleaved into 2 fragments such that exon 3 is separated from the residual portion of intron 3.

Point mutations (single-nucleotide substitutions) were the most common gene defects. Few mutations occur in the introns of the F9 gene, while most insertions and deletions in the exons cause frame shifts, leading to severe HB in almost all patients [8]. The findings of the present study further clarify the pathogenesis of HB, although the mechanism by which mutations in the F9 gene lead to dyspoiesis remains to be elucidated. Although further studies in this pedigree are required, this study provides the basis of a method for prenatal diagnosis of HB. 

**Acknowledgments**

We thank all the patients and their families who participated in this study. This study was supported by the National Foundation of China (No. 30973140), the Doctor Starting Funding of Liaoning (No. 20111123), and the Natural Science Foundation of Liaoning (No. 201202230).

**Conflict of Interest Statement**

The authors of this paper have no conflicts of interest, including specific financial interests, relationships, and/or affiliations relevant to the subject matter or materials included.

## Figures and Tables

**Table 1:**
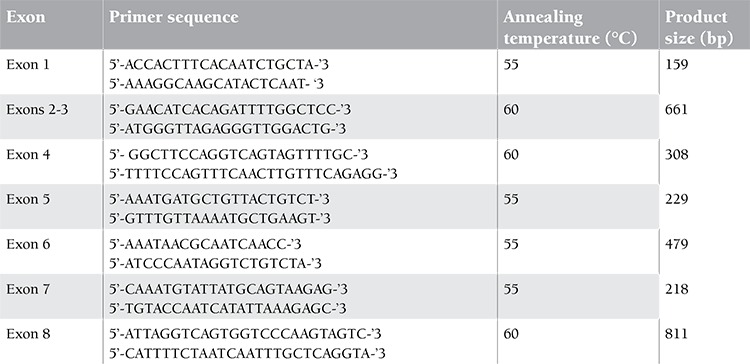
Primers for mutation analysis of factor IX gene.

**Table 2:**
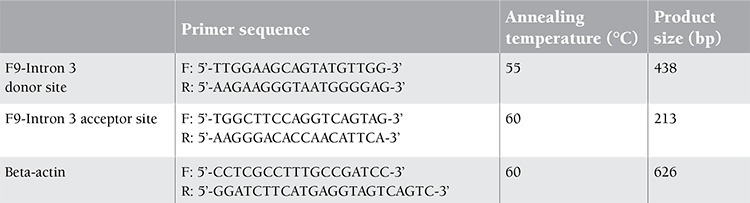
RT-PCR primer details.
